# A Scientometric Evaluation of COVID-19 and Male Reproductive Research

**DOI:** 10.3390/clinpract13060118

**Published:** 2023-10-26

**Authors:** Manesh Kumar Panner Selvam, Anika Kapoor, Saradha Baskaran, Ajaya Kumar Moharana, Suresh C. Sikka

**Affiliations:** 1 Department of Urology, School of Medicine, Tulane University, New Orleans, LA 70112, USA; saradhabaskaran@gmail.com (S.B.); amoharana@tulane.edu (A.K.M.); 2 The Hockaday School, Dallas, TX 75229, USA; akapoor25@hockaday.org; 3 Redox Biology & Proteomics Laboratory, Department of Zoology, School of Life Sciences, Ravenshaw University, Cuttack 753003, Odisha, India

**Keywords:** SARS-CoV-2, COVID-19, scientometrics, male reproduction, male infertility

## Abstract

The COVID-19 pandemic due to the SARS-CoV-2 coronavirus showed acute and prolonged effects on human health. In addition, over the past four years, there has been a tremendous surge in COVID-19-related scientific publications, as shown by bibliometric and scientometric studies. However, such analysis of the scientific literature is lacking in the area of male reproduction. The current scientometric study analyzes publication characteristics of articles related to male reproduction and COVID-19 infection. We used the Scopus database to analyze scientometric data (the number of publications, journals, countries, type of documents, and subject area) related to COVID-19 and male reproductive research. Our literature search identified 345 articles related to COVID-19 and male reproductive research. Most of the articles were published in the USA (*n =* 72), Italy (*n =* 55), and China (*n =* 51). Such research was mainly focused around medicine (57.1%), followed by biochemistry, genetics, and molecular biology (25.7%). Also, in the area of male reproduction, only 37.1% (*n =* 128) of the articles contributed towards original research, whereas 52.8% (*n =* 182) were review articles and editorials focusing more on sexual dysfunction than infertility. Such a small number of studies published on COVID-19-related effects on male reproduction warrants a significant increase in research, which is required to decipher the mechanism(s) underlying SARS-CoV-2 infection-associated impairment of male reproductive function.

## 1. Introduction

The coronavirus disease (COVID-19), caused by the SARS-CoV-2 virus, has resulted in nearly 15 million deaths worldwide [1]. The COVID-19 pandemic has also resulted in significant public health, social, and economic impacts worldwide [2,3]. SARS-CoV-2 infection mainly causes respiratory, digestive, cardiovascular, urinary, and nervous system complications [4,5]. The virus gains entry into cells by attaching itself to specific angiotensin-converting enzyme 2 (ACE2) receptors that are present on the surface of the cells [6] of various tissues (such as the respiratory tract, blood vessels, heart, intestines, and kidneys), including the reproductive system [7]. Male reproductive organs contain a substantially greater number of ACE2 receptors than female reproductive system organs [8,9]. Because of the ample expression of ACE2 receptors in the penis (especially corpus cavernosum) and testis, the male reproductive system is vulnerable to the effects of COVID-19 infection, thus affecting the sexual health and fertility of men [10,11,12].

Globally, infertility impacts more than 80 million couples (i.e., 15%) in the reproductive age group. The male factor is responsible for 50% of such infertile cases [13,14]. In general, laboratory evaluation of male infertility typically involves semen analysis mainly to assess sperm count, total motility, and morphology, in addition to a detailed physical, medical, and sexual history evaluation, including examination of the testes [13,14,15]. Combined, these can provide accurate information on a man’s fertility potential [16]. On the other hand, male sexual dysfunction includes difficulties with sexual desire, arousal, erection, orgasm, or ejaculation [17]. Both male infertility and sexual dysfunction can significantly impact not only a man’s but a couple’s quality of life and emotional well-being [18,19]. Research on male infertility and sexual dysfunction is continuously evolving to understand better the causes, risk factors, and treatment options for these conditions. Recent reports revealed that the male reproductive system is directly and indirectly affected by SARS-CoV-2, thus compromising its fertility potential [7,20]. The latest research trends in male infertility and sexual health have been analyzed and reported using bibliometric and scientometric tools [21,22,23,24]. However, there is scant information and publications on research trends on how COVID-19 is linked to male reproductive health.

Scientometrics is an interdisciplinary field involving quantitative analysis of science and scientific research [25]. It uses various bibliometric and statistical methods to study different aspects of scientific publications, such as journals, articles, authors, citations, research institutions, and scholarly communication [25,26]. These are essential tools used to evaluate and assess research productivity and scientific impact. It also provides valuable insights into the dissemination of knowledge, research trends, and the impact of scholarly work. Researchers frequently make efforts to quantify the development of a scientific field, the significance of peer-reviewed academic publications, and authorship patterns. Scientometrics is an important tool that has been widely used to study and analyze the scientific literature [27,28,29,30,31,32]. Since the onset of the COVID-19 pandemic and frequent occurrences of new variants along with “long COVID” effects, such scientometric tools are providing meaningful insight and gaining importance for current and future research in the medical field. However, no scientometric analysis studies are available to date that specifically focus on COVID-19 research in the area of male reproduction. The current scientometric study identifies and analyzes publication characteristics of articles on male reproduction and COVID-19.

## 2. Materials and Methods

### 2.1. Data Source

Scopus is a thorough bibliographic database that covers 17.6 million author profiles, and more than 87 million documents with 1.8 billion cited references [33]. Scopus provides details such as the type of document, number of citations, number of documents per year, authors and their affiliation, journal, country, and subject area, as required for scientometric analysis. In the current study, we used this Scopus database to identify the publications associated with male reproduction and COVID-19.

### 2.2. Data Retrieval Strategy

**Figure 1:**
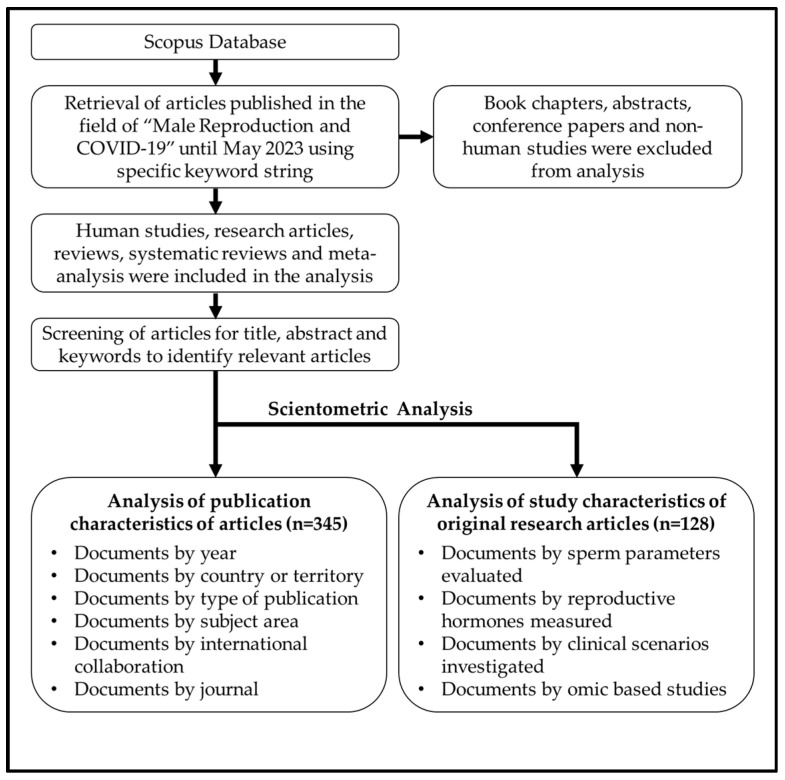
The **f** low diagram signifiesthe data retrieval strategy for our scientometric analysis.

For a search of the literature in the Scopus database, we used a specific set of keywords (Table 1) to retrieve the articles published on male reproduction and COVID-19 (Figure 1). We limited our search to scientific articles published only on human subjects. Using the asterisk ‘*’ mark after a word, we included all possible variants of the term along with multiple Boolean operators such as ‘AND’, ‘OR’, ‘NOT’, and ‘AND NOT’ to invalidate false positive results. Also, functions such as ‘TITLE-ABSTRACT’ and ‘TITLE-ABSTRACT-KEYWORDS’ were used to retrieve a maximum number of relevant articles. Animal studies, non-English articles, and publications unrelated to male reproduction and COVID-19 were excluded from this analysis.

Any relevant article was categorized based on publication type (research or review article), subject area, and journal type. Further, we have sorted the original research articles according to clinical scenarios linked to male reproductive disorders or dysfunction, i.e., we classified the original studies that reported sperm parameters and reproductive hormone levels in SRAS-CoV-2 infected men in this analysis.

### 2.3. Scientometric and Statistical Analysis

The scientometric data that we retrieved from Scopus as comma-separated value (CSV) files was converted to Microsoft Excel data files for the purpose of descriptive statistical analysis. Such CSV files related to COVID-19 and male reproductive research were then used for geographical mapping. Further, we generated network maps based upon international collaboration in the area of COVID-19 and male reproductive research by using the software “VOSviewer” (downloaded from http://vosviewer.com, accessed on 11 June 2023) [34]. The number of documents co-authored by investigators from different countries was used to measure the relatedness between the countries. Whereas the size of the node was determined by the number of articles published by each country.

## 3. Results

### 3.1. COVID-19 and Male Reproductive Research

**Figure 2:**
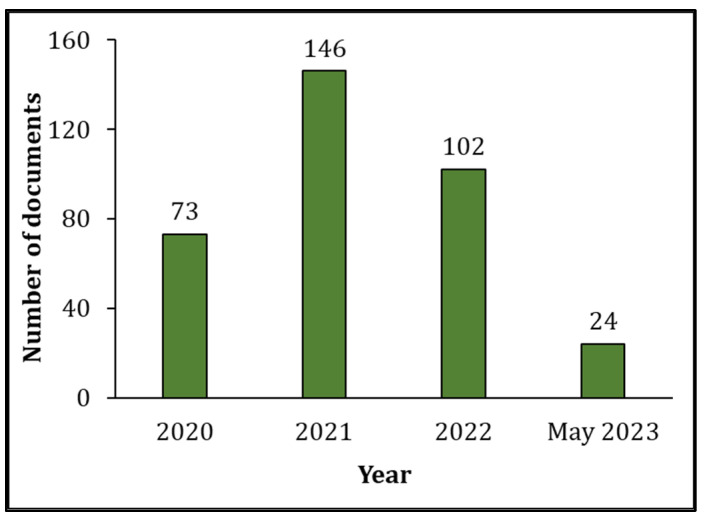
Number of publications per year since the COVID-19 pandemic (2020 to May 2023) related to COVID-19 and male reproductive research.

**Figure 3:**
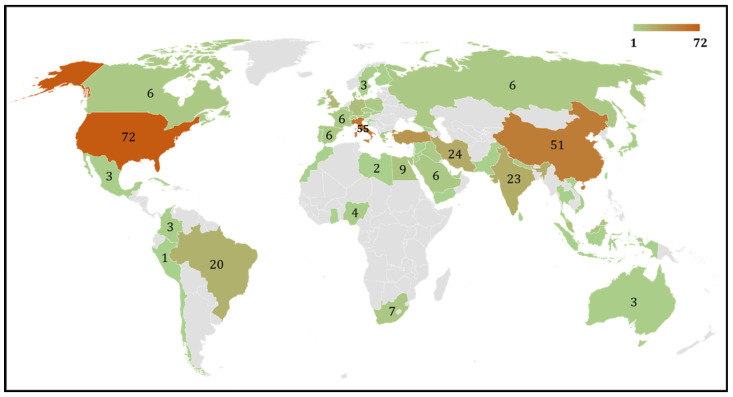
Geomap depicting the distribution of number of publications from various countries that contributed to this research related to COVID-19 and male reproduction.

**Figure 4:**
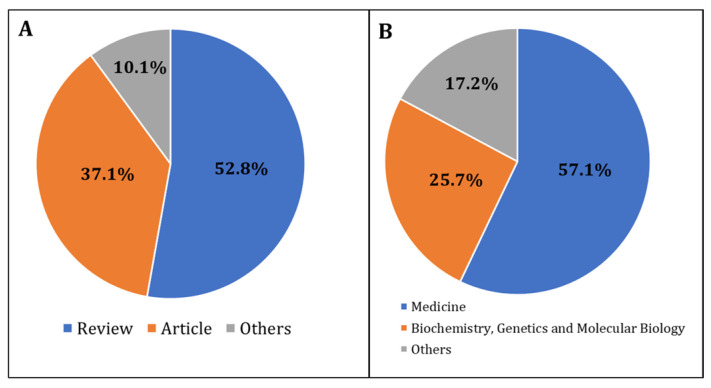
Research trends based on the (**A**) type of publications, and (**B**) study areas that were related to COVID-19 and male reproduction during the period 2020 to May 2023.

**Figure 5:**
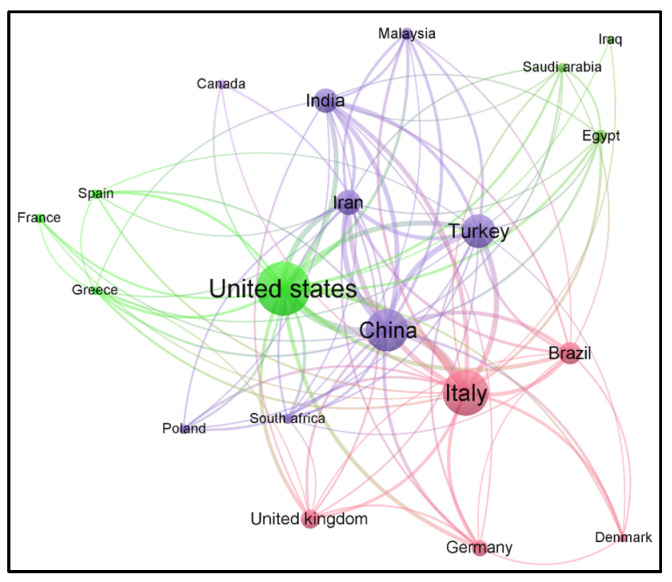
Network map showing various countries that published such articles in the field of research related to COVID-19 and male reproduction from 2020 to May 2023.

**Figure 6:**
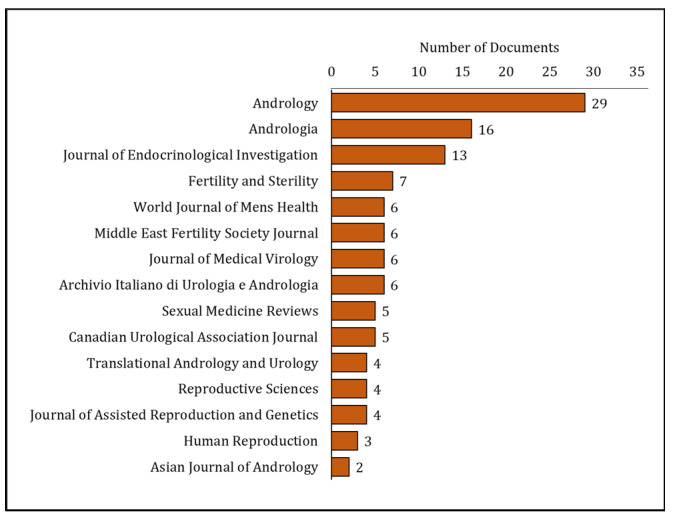
Various journals that published research on COVID-19 and male reproduction from 2020 to May 2023.

Our search of the literature showed 345 research articles that were directly related to COVID-19 and male reproduction (Figure 2). The majority of these articles were published in the USA (*n =* 72), then Italy (*n =* 55), and China (*n =* 51) (Figure 3). The majority of these publications were in the form of review articles (*n* = 182, 52.8%), and fewer were original articles (*n* = 128, 37.1%) (Figure 4A). The main research was in the fields of medicine, genetics and molecular biology, biochemistry, and microbiology/ immunology (Figure 4B). The network map shown in Figure 5 suggests collaboration between the countries. The major journals that published articles in this area were Andrology (*n* = 29), Andrologia (*n* = 16), and the Journal of Endocrinological Investigation (*n* = 13) (Figure 6).

### 3.2. The Scientometrics of COVID-19: Semen Parameters, Reproductive Hormones, and Viral Tropism Studies

Our publication trend analysis revealed that among the 128 research articles, 38 studies assessed male reproductive hormones (such as testosterone, luteinizing hormone, follicle-stimulating hormone, prolactin, estradiol, and inhibin) in COVID-19 patients with reproductive issues. In addition, 37 studies evaluated the effect of COVID-19 on semen parameters.

Scientometric analysis revealed that semen (*n =* 24) was the most common male reproductive system biospecimen to detect SARS-CoV-2 from COVID-19 patients. Other specimens, such as testis (*n =* 3) and penile tissue (*n =* 1), were also evaluated for the SARS-CoV-2 virus. Further, publication trend analysis revealed that 16.4% of studies (*n =* 21) investigated the expression of ACE receptors in sperm or testicular tissues. 

### 3.3. COVID-19 Pandemic: Clinical Scenarios Associated with Men’s Sexual Health and Infertility

We investigated research publications on COVID-19 and male reproduction under different clinical situations related to male reproduction (Table 2). Our results showed that most of the COVID-19-related studies were focused on the area of erectile dysfunction (*n =* 21) followed by evaluation of semen characteristics (*n =* 6), varicocele (*n =* 5), idiopathic infertility (*n =* 2) and orchiepididymitis (*n =* 1). Our analysis revealed that erectile dysfunction was the leading area that gained more attention in COVID-19 and male reproduction research. Furthermore, we also noticed that 23 studies (18%) investigated the histopathology and pathophysiology of the male reproductive system associated with COVID-19 infection. In addition, only three studies investigated seminal oxidative stress-induced sperm DNA fragmentation in SARS-CoV-2-infected men.

### 3.4. Publication Trends in Omics-Based Male Reproductive Research during COVID-19 Infection

Finally, we evaluated publication trends on the impact of SARS-CoV-2 on molecular changes in male reproductive organs. Scientometric analysis revealed that ten studies (7.8%) used omics platforms to study the transcriptome and proteome of male reproductive organs.

## 4. Discussion

The COVID-19 pandemic, on a global scale, negatively affected the wellness of human beings, including their reproductive health [7]. Several scientometric, bibliometric, and publication pattern studies were published on COVID-19 in various fields of biomedical research [32,35,36,37,38,39,40]. Scientometric studies provide a global evaluation report of scientific publications on a specific topic. This study is the first methodical evaluation of COVID-19 and male reproductive health based on a scientometric approach. The literature analysis identified the extent of publications on COVID-19 related to male reproductive research, with a maximum number of studies in 2021. Interestingly, publications on COVID-19 and male reproduction were mostly review articles compared to original research articles. Geo-mapping and network analysis clearly illustrated the USA as the top publishing country studying the impact of this virus on men’s sexual health and infertility, along with its international collaboration with other countries. 

SARS-CoV-2 enters the cells via the ACE2 receptor, which is also present in different organs, including the testes [6]. Further, SARS-CoV-2 can activate the ACE2 receptor in seminal plasma components, making semen a transmission route to COVID-19 infection [41]. Our scientometric results indicated that 16.4% of studies investigating the consequences of COVID-19 on male reproduction measured the expression of the ACE2 receptor in sperm or testicular tissues. Mainly, the ACE2 receptors are expressed in the sperm midpiece and distributed in the acrosomal region of the sperm head. Whereas in human testis, ACE2 receptors are highly expressed in spermatogonia, Leydig, and Sertoli cells [42]. Interestingly, SARS-CoV-2 was also detected in the penile tissue of infected patients, even those who recovered from this infection [43,44]. In addition, SARS-CoV-2 has been identified in other body fluids such as saliva, blood, urine, and cerebrospinal fluid [45,46]. We noticed that about 18.8% of studies published on the effects of COVID-19 on male reproduction screened semen samples for the presence of the virus. Several studies have documented the shedding of SARS-CoV-2 in the semen of men with symptomatic and asymptomatic phases [47,48]. In contrast, other investigators were unable to detect virus particles in the semen of men infected with SARS-CoV-2 [49,50,51]. Hence, the detection of viruses in semen samples remains debatable. It may be possible that the strain or variant and virulence factor of the virus may have a crucial role in causing damage to the blood–testis barrier (BTB) and other accessory reproductive glands, such as seminal vesicles and the prostate, and thus infiltrate into semen. Additional studies are required to draw a meaningful conclusion to validate the transmission of SARS-CoV-2 through semen.

COVID-19 infection has been linked to male infertility [7], and molecular findings revealed defective spermatogenesis and an impaired BTB in infected men [52,53]. The scientometric analysis indicated that nearly 28.9% of studies focused on reproductive health investigated the semen parameters of SARS-CoV-2-infected men. Also, recent meta-analysis results reported a negative association between semen quality and SARS-CoV-2 infection [54,55,56,57]. However, till now, none of the studies investigated the long-term impact of this virus on semen quality and reproductive function. Few studies have focused on male infertility associated with semen abnormalities (4.7%), varicocele (3.9%), and idiopathic infertility (1.6%), evaluating the repercussions of this disease on sperm parameters. Systemic viral infection negatively impacts sperm production and the quality of semen [58]. Further, such viral infections may induce damage to sperm DNA, impairing their ability to fertilize oocytes [59,60]. Based on our current analysis in this area, very few studies (*n =* 3) reported seminal oxidative stress and damage to sperm DNA induced by SARS-CoV-2 infection. Seminal oxidative stress was evaluated based on the increased levels of reactive oxygen species (ROS) or decreased antioxidant levels in the semen or seminal plasma, whereas sperm DNA fragmentation is commonly measured based on the extent of chromatin dispersion (halos) observed under the microscope. A positive correlation exists between oxidative stress and sperm DNA damage [61]. However, the literature still lacks mechanistic studies that can delineate the negative influence of SARS-CoV-2 infection on sperm DNA and fertility. 

Scientometric analysis indicated nearly 29.7% of research articles reported reproductive hormone levels in SARS-CoV-2-infected men. The hypothalamus-pituitary-gonad (HPG) axis maintains the balance of male sex hormones and gonadal function [62,63]. SARS-CoV-2 infection negatively impacts the HPG axis, affecting male reproductive hormone levels and testicular physiology [64,65]. Endocrine disturbances linked to the male reproductive system decrease testosterone levels in patients with COVID-19, resulting in hypogonadism, which may result in erectile dysfunction (ED) [66,67]. In addition, other hormones, such as LH and FSH levels, were significantly lower, and estradiol levels were higher in COVID-19 patients [68,69]. Whereas no such change was reported in the prolactin levels. Our analysis also shows that research on male reproductive health is focused more on ED. Men infected or recovered from SARS-CoV-2 infection are more prone to ED issues than uninfected men [70,71]. However, additional research is required to establish a causal link between COVID-19, hypogonadism, and ED. Further, the exact mechanisms and the extent of this impact are not yet fully understood.

Omics platforms, such as genomics, transcriptomics, and proteomics, including single-cell RNA sequencing techniques, are widely employed to understand the changes at the subcellular level due to infection [72]. Most of the omics studies on SARS-CoV-2 infection and its consequence on testis focused on understanding the expression pattern of ACE2 in various types of cells. Transcriptomic analysis revealed the negative impact of COVID-19 on spermatogenesis and the dysregulation of molecular pathways linked to the immune response, which in turn may alter spermatogenesis by inducing testicular cell senescence through the MAPK signaling pathway [73]. Further, impaired spermatogenesis was supported by histopathological changes such as the presence of degenerated germ cells (GCs) in the lumen of seminiferous tubules that indicated negative a impact of SARS-CoV-2 infection on GC development [74]. In addition, proteomic analysis revealed downregulation of proteins involved in reproductive function in the seminal plasma of COVID-19-recovered patients, indicating that the fertility potential of these men was compromised post-SARS-CoV-2 infection [75]. However, further studies are needed to understand the impact of long COVID-19 on spermatogenesis, semen quality, and overall impact on male reproduction, especially since many new variants are frequently evolving.

In the present study, we have analyzed the data of the last four years, thus including the most recent publications (2019–May 2023) since COVID-19 was declared a pandemic. Thus, it represents all the publications available on COVID-19 and male reproductive research. However, we have only used bibliometric information retrieved from the Scopus database, and therefore, a few publications not indexed in Scopus could have been omitted from the analysis.

## 5. Conclusions

To our knowledge, this is the first scientometric analysis showing a detailed assessment of published studies focused on COVID-19 and male reproduction. Our analysis was focused on both male infertility and sexual dysfunction studies. This information is useful to researchers interested in the latest developments in this area and looking forward to evidence-based research concerning the COVID-19 pandemic. A small number of studies have been published on male reproduction compared to other fields related to the health sciences. A significant increase in COVID-based reproductive research is required to ascertain the mechanism(s) underlying SARS-CoV-2 infection-associated impairment of male reproduction and the impact of long COVID-19.

## Figures and Tables

**Table 1:** Search terms or keywords used to identify relevant articles related to male reproduction and COVID-19.

Scopus (Search Date: 31 May 2023)
TITLE-ABS-KEY(“COVID”OR“SARS-CoV-2”OR“COVID-19”OR“COVID-19”OR“pandemic”)ANDTITLE-ABS-KEY(“male reproducti*”OR“male fertility”OR“male infertility”OR“male subfertility”OR“male sterility”OR“seminal plasma”OR“semen”OR“seminal fluid”OR“sperm*”OR“testis”OR“testicular”OR“gonad*”OR“testosterone”OR“prostat*”OR“epididy*”OR“erectile dysfunction”OR“ejaculat*”)

**Table 2:** Clinical scenarios coupled with men’s sexual health and infertility investigating the effect of COVID-19 on male reproduction during the years 2020 to May 2023.

Clinical Scenarios	Number of Studies (n)	% of Studies Evaluated
Erectile dysfunction	21	16.4%
Semen abnormalities	6	4.7%
Varicocele	5	3.9%
Idiopathic infertility	2	1.6%
Orchiepididymitis	1	0.8%

## Data Availability

Not applicable.
